# Influence of monoterpenoids on the growth of freshwater cyanobacteria

**DOI:** 10.1007/s00253-021-11260-8

**Published:** 2021-06-23

**Authors:** Lucyna Balcerzak, Stanisław Lochyński, Jacek Lipok

**Affiliations:** 1 Department of Chemical Biology and Bioimaging, Faculty of Chemistry, Wroclaw University of Science and Technology, Wyb. Wyspiańskiego 27, 50-370 Wrocław, Poland; 2 Institute of Cosmetology, Wrocław College of Physiotherapy, Wrocław, Poland; 3 Department of Pharmacy and Ecological Chemistry, Faculty of Chemistry, Opole University, Opole, Poland

**Keywords:** Cyanobacteria, Monoterpenoid, Inhibition of growth, Bloom formation, Cyanobacterial consortium

## Abstract

**Abstract:**

Cyanobacteria are characterized by a very high tolerance to environmental factors. They are found in salt water, fresh water, thermal springs, and Antarctic waters. The wide spectrum of habitats suitable for those microorganisms is related to their particularly effective metabolism; resistance to extreme environmental conditions; and the need for only limited environmental resources such as water, carbon dioxide, simple inorganic salts, and light. These metabolic characteristics have led to cyanobacterial blooms and the production of cyanotoxins, justifying research into effective ways to counteract the excessive proliferation of these microorganisms. A new and interesting idea for the immediate reduction of cyanobacterial abundance is to use natural substances with broad-spectrum biological activity to restore phytoplankton diversity. This study describes the effects of selected monoterpenoid derivatives on the development of cyanobacterial cultures. In the course of the study, some compounds ((±)-citronellal, (+)-α-pinene) showed the ability to inhibit the colonization of the tested photosynthetic bacteria, while others (eugenol, eucalyptol) stimulated the growth of these microorganisms. By analyzing the results of these experiments, information was obtained on the mutual relations of cyanobacteria and the tested monoterpenes, which are present in the aquatic environment.

**Key points:**

*• Monoterpenoids significantly inhibit the growth of single cyanobacterial strains.*

*• Monoterpenoids can inhibit the growth of cyanobacterial consortia.*

*• Natural substances can control the growth of freshwater cyanobacteria.*

**Supplementary Information:**

The online version contains supplementary material available at 10.1007/s00253-021-11260-8.

## Introduction

Cyanobacteria in principle require only water, carbon dioxide, inorganic substances, and light to live (Fay 1965; Vioque 2007). These limited environmental requirements make blue-green algae pioneer microorganisms; they occur most frequently in freshwater and marine environments but can also be found in damp soil, in hot springs, on bare rock, on soil, and even on Antarctic rocks (Rothschild and Mancinelli 2001; Alwathnani and Johansen 2011; Schopf 2012; Waajen et al. 2014). Moreover, blue-green algae show a very high tolerance to environmental factors and are known to form water blooms in response to even slightly better growth conditions. Among the main causes of blooms are high concentrations of phosphorus and nitrogen (Harke et al. 2012; Jiang et al. 2014). Additionally, many other factors, including light intensity, pH, temperature, and turbulence, influence bloom formation (de Figueiredo et al. 2004; de Souza Santos et al. 2011; Dai et al. 2012; Soares et al. 2013). The main species responsible for problems with bloom toxicity is *Microcystis aeruginosa.* These cyanobacteria commonly occur in Europe, Asia, and North America (Bláhová et al. 2013). Kormas et al. 2011 indicated the presence of this species among others, which create typical cyanobacterial consortia; these species include *Aphanocapsa incerta*, *Chroococcus limneticus*, *Microcystis flos-aquae*, *Microcystis aeruginosa*, *Snowella lacustris*, *Synechococcus* spp., *Limnothrix redekei*, *Jaaginema* sp., *Oscillatoria* sp., *Planktolyngbya circumcreta*, *Planktothrix agardhii*, *Anabaena flos-aquae*, *Anabaena aphanizomenoides*, and *Aphanizomenon issatshenkoi* (Kormas et al. 2011). Some other species responsible for bloom formation include *Cylindrospermopsis raciborskii* in tropical regions (Sinha et al. 2012) and *Planktothrix* sp*.* in Europe and South America (Bonilla et al. 2012).

A massive and serious environmental problem is cyanobacterial water bloom as a result of eutrophication (O’Neil et al. 2012; Paerl and Paul 2012). The formation of cyanobacterial blooms in fresh water is a serious problem in the management of drinking water and causes odors in the water during the summer months, thus reducing the attractiveness of tourist destinations (Heisler et al. 2008; Dai et al. 2012). Many species produce toxic compounds such as neurotoxins (homoanatoxin-a, anatoxin-a, anatoxin-a(s), and saxitoxins) (Codd 2000), hepatotoxins (cylindrospermopsin, nodularin, and microcystins), and dermatotoxins (lipopolysaccharides, aplysiatoxins, and lyngbyatoxin-a) (Dawson 1998; Rastogi and Sinha 2009; Kormas et al. 2011; Bittencourt-Oliveira et al. 2012; O’Neil et al. 2012). Cyanobacterial toxins are dangerous for people and other animals since they may cause staggering, hypersalivation, muscle fasciculations, gasping, liver damage, skin irritation, or muscle paralysis (Codd 2000).

Currently, there are several methods to remove cyanotoxins from drinking water, including activated carbon, slow sand filtration, ozonation, membrane filtration, coagulation, chlorination, UV irradiation of water, and bioaccumulation of MC-LR (microcystin-LR) in aquatic macrophytes (Jurczak et al. 2005; Sharma et al. 2012). Some research has been conducted on the inhibition of cyanobacterial growth by nanocrystals (Fan et al. 2019), riparian tree leaf extract (Le Rouzic et al. 2016), and Cu-nanoparticle-embedded biochar composite (Li et al. 2019). However, these methods of water treatment possess serious disadvantages: they are very expensive, require appropriate equipment, may have low efficiency, require lengthy treatment, and very often are not available to poor countries or people with ponds (Nimptsch et al. 2008). Thus, there is a real need to find a safer, more effective, and economically accepted method to control harmful cyanobacterial blooms.

In our experiments, the influence of selected monoterpenoids on the growth of freshwater cyanobacteria was studied to verify how these natural substances interact with blue-green algae. Monoterpenoids compose the largest and most structurally diverse class of terpenes (Degenhardt et al. 2009; Groussin and Antoniotti 2012). Most of these substances are common in nature as plant metabolites; additionally, some monoterpenes are cheap and readily available, as they are often considered byproducts of the food, pharmaceutical, or cosmetic industries (Groussin and Antoniotti 2012). These attributes, together with their well-known biological activity, make monoterpenoids ideal substrates for experiments to investigate their influence on the growth of cyanobacteria.

Specifically, in this article, we show the influence of citral, (±)-citronellal, (±)-citronellol, eugenol, (+)-carvone, (+)-dihydrocarvone, (+)-α-pinene, eucalyptol, and (+)-3-carene (Fig. S1) on the growth of *Anabaena* sp., *Chroococcus minutus*, and *Nodularia moravica*.

## Materials and methods

### Test organisms and culture conditions

Strains of the cyanobacteria *Anabaena* sp. PCC 7937 TISCHER/UTEX 1444 (strain CCALA 007), *Chroococcus minutus* HINDAK 1969/23 (strain CCALA 055), and *Nodularia moravica* HINDAK 2000/17 (strain CCALA 797) were obtained from the Institute of Botany, Academy of Sciences of the Czech Republic. Cultures were grown in BG11 medium (ATCC 616) at a temperature of 24±1 °C and a photoperiod of 16–8 light–dark with a light intensity of 300 μmol·m^−2^·s^−1^.

### Survival cultures

Survival cultures were made in sterile 250-mL Erlenmeyer flasks containing 50 mL of BG11 medium (ATCC 616). Cultures were revitalized every 21 days by transferring 10-mL aliquots to fresh medium.

### Measurement of total chlorophyll content

The determination of the growth of the examined photoautotrophs was performed using time-course measurements of total chlorophyll content in experimental cultures. Therefore, at 3–4-day intervals, growth was followed by harvest as follows: 1 mL of solution was taken from each culture, and the cells were sedimented via centrifugation (5 min, 13,000×*g*). The 0.9-mL volumes of supernatant were removed from the samples, and the remaining cells were resuspended in 0.9 mL of methanol. The samples were shaken for 20 s and placed in darkness. After 10 min, the content was shaken again for 20 s and placed in darkness. After 10 more minutes, the samples were centrifuged as above, and total chlorophyll content in the supernatant was determined spectrophotometrically on the basis of Arnon’s formula-total chlorophyll [*a*+*b*]=20.21·*E*_645_+8.02·*E*_663_ (Porra 2002) using a Hitachi (Tokyo, Japan) U 2810 spectrophotometer. The average chlorophyll levels in each replicate of the experimental or control culture correlated with the day of the experiment and were plotted based on a time course, yielding growth curves.

### Experimental cultures

The experimental cultures were prepared by transferring appropriate volumes of aliquots from 3-week-old subcultures to fresh media. In this case, the experimental cultures were concentrated via centrifugation (1 min, 5000 rpm). Then, total chlorophyll content was measured. The volumes of the inocula for each strain were established experimentally based on the final chlorophyll concentration, which was 1 μg/mL. Experimental cultures were prepared in 250-mL Erlenmeyer flasks by adding appropriate amounts of each substrate to reach final concentrations of 0.2, 0.4, 0.6, 0.8, and 1 mM in 50 mL of culture containing 1 μg of chlorophyll per 1 mL of volume in medium. To obtain a homogenous dispersion of hydrophobic monoterpenoids in the aqueous microbial cultures, the examined compounds were dissolved in 0.15 mL of acetone—this volume of acetone was experimentally proven to not influence the growth of cyanobacteria—and then added to the culture. Cultures containing only acetone and no monoterpenoids served as controls.

All experiments or controls had at least three repetitions. The influence of monoterpenoids on the growth of the examined cyanobacteria was determined via time-course measurements of total chlorophyll content in experimental cultures in relation to the appropriate control. Therefore, total chlorophyll content was measured at 3–4-day intervals. Cultures were grown for 2 weeks, and the results of the experiments are presented in the graphs versus various concentrations of the tested monoterpenoids.

A mixture of three species of freshwater cyanobacteria and one selected isoprenoid derivative was used to determine the sensitivity of the cyanobacterial consortium to the monoterpenoid. For this purpose, experimental cultures were prepared as described above except that the volumes of cyanobacterial inocula placed in the flask were added such that each species composed 1/3 of the final chlorophyll concentration of 1 μg/mL. On the last day of the experiment, microscopic observations were made of the growth media.

To investigate the ability of cyanobacteria to biotransform monoterpenoids, experimental cultures were prepared as described above, and the final substrate concentration in the culture was 1.0 mmol/L. The controls contained only the medium with dissolved monoterpenoid. The cultures were kept for 14 days in a room with constant lighting and temperature conditions. After 2 weeks, the contents of each flask were extracted 3 times with 25 mL of dichloromethane or chloroform. The organic phase was dried over anhydrous magnesium sulfate, and the solution was concentrated on a rotary evaporator. The obtained samples were subjected to TLC analysis and gas chromatography coupled to mass spectrometry (Hewlett-Packard 6890 Series coupled with high-temperature MS Hewlett-Packard 5073 with a 30 m × 0.32 mm HP-5 capillary column) or GC-FID (Thermo Scientific with an HP-5 capillary column). The standard temperature program was (a) 40 °C for 5 min, (b) 10.0 °C/min from 40 to 190 °C and hold temperature for 5 min, and (c) 20.0 °C/min from 190 to 250 °C and hold temperature for 10 min.

## Results

Our studies verified an influence of selected monoterpenoids, including citral, (±)-citronellal, (±)-citronellol, eugenol, (+)-carvone, (+)-dihydrocarvone, (+)-α-pinene, eucalyptol, and (+)-3-carene, on the growth of the freshwater cyanobacteria species *Anabaena* sp., *Chroococcus minutus*, and *Nodularia moravica*.

### The influence of single monoterpenoids on the growth of cyanobacteria

**Fig. 1:**
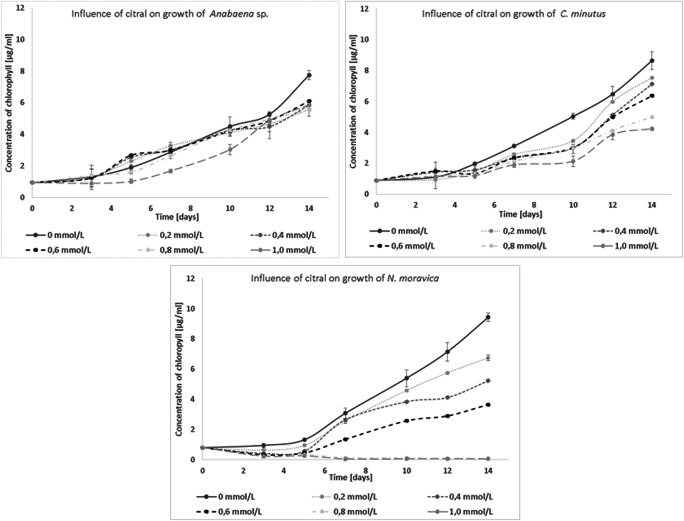
Influence of citral on the growth of freshwater cyanobacteria

*N. moravica* was the most sensitive cyanobacterial species to the presence of citral; in this experiment, after 3 days, no growth was observed with citral concentrations of 0.8 and 1.0 mmol/L. The growth of the other tested species was also limited by citral; however, *Anabaena* sp. and *C. minutus* grew even with the highest concentration of citral (Fig. 1).

**Fig. 2:**
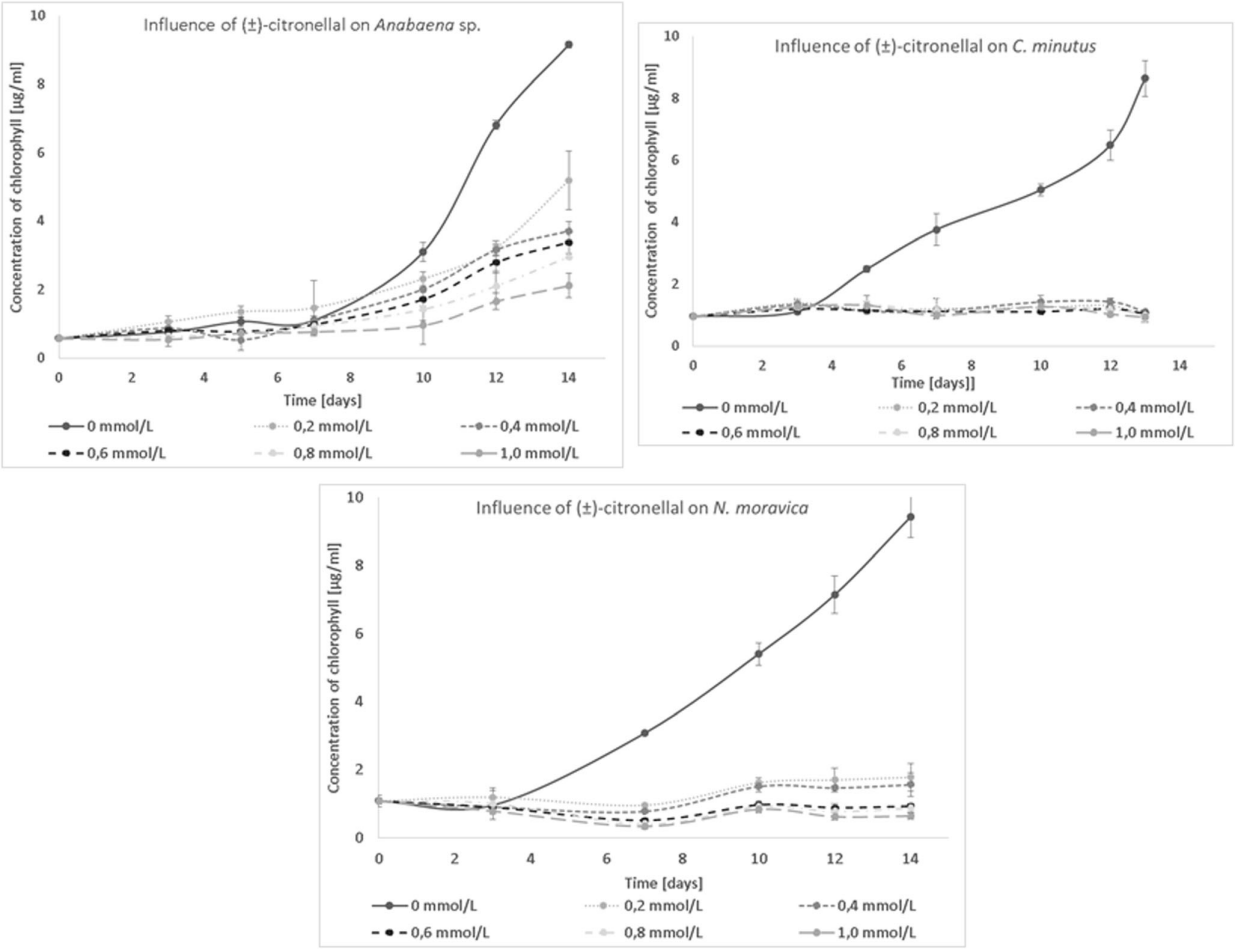
Influence of (±)-citronellal on the growth of freshwater cyanobacteria

All tested species of blue-green algae were characterized by a similar type of sensitivity to the presence of (±)-citronellal. *C. minutus* and *N. moravica* showed limited growth in the early days of culturing. Consequently, the concentration of chlorophyll remained at 1 μg/mL until the last day of the experiment, the fourteenth (14th) day. The development of *Anabaena* sp. was also limited by (±)-citronellal, but at the lower tested concentration of this monoterpenoid, the chlorophyll content remained above 5 μg/mL (Fig. 2).

**Fig. 3:**
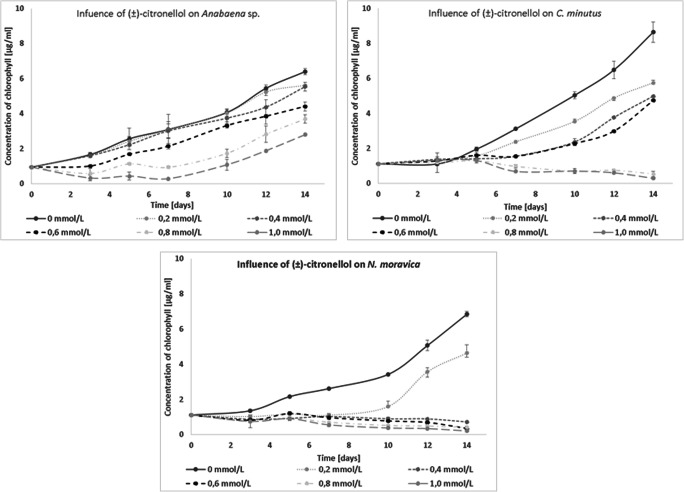
Influence of (±)-citronellol on the growth of freshwater cyanobacteria

All tested blue-green algae were limited by (±)-citronellol. *N. moravica* was so sensitive that on the last day of culture, very little development was observed with almost all tested concentrations of this compound; only with 0.2 mmol/L of (±)-citronellol was growth visible. *C. minutus* was also limited by this compound; only with 0.2, 0.4, and 0.6 mmol/L of (±)-citronellol was growth observed (Fig. 3).

**Fig. 4:**
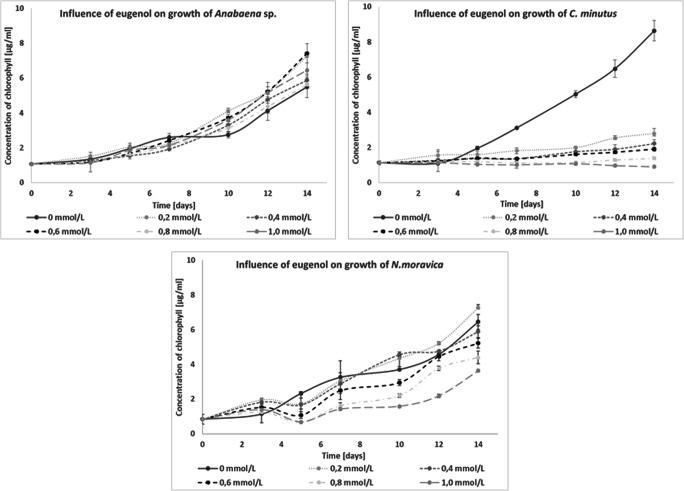
Influence of eugenol on the growth of freshwater cyanobacteria

The presence of eugenol in the *C. minutus* culture caused significant inhibition of development with all tested concentrations. *N. moravica* also showed limited growth, but when lower concentrations of eugenol were used, more development was observed than in the control. On the other hand, *Anabaena* sp. showed increased growth with all tested concentrations of this compound compared with the control (Fig. 4).

**Fig. 5:**
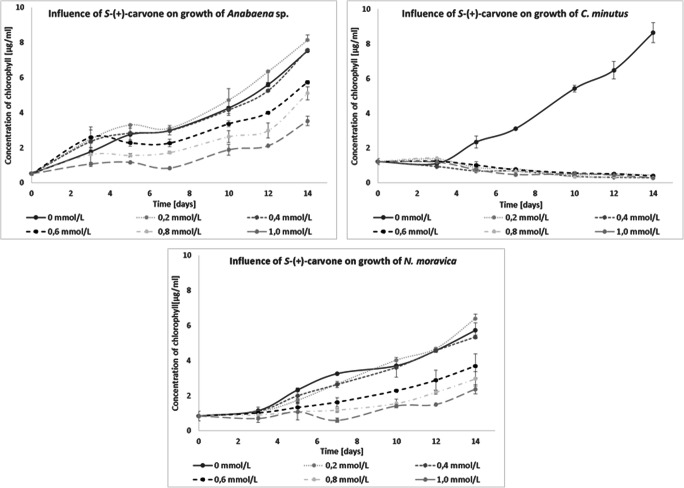
Influence of (+)-carvone on the growth of freshwater cyanobacteria

**Fig. 6:**
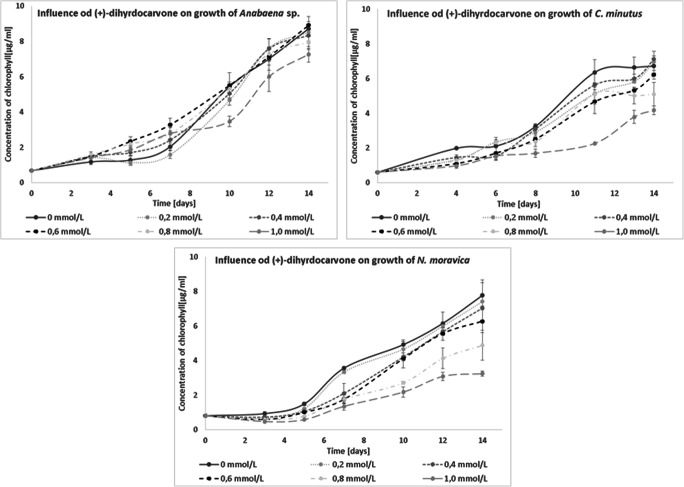
Influence of (+)-dihydrocarvone on the growth of freshwater cyanobacteria

**Fig. 7:**
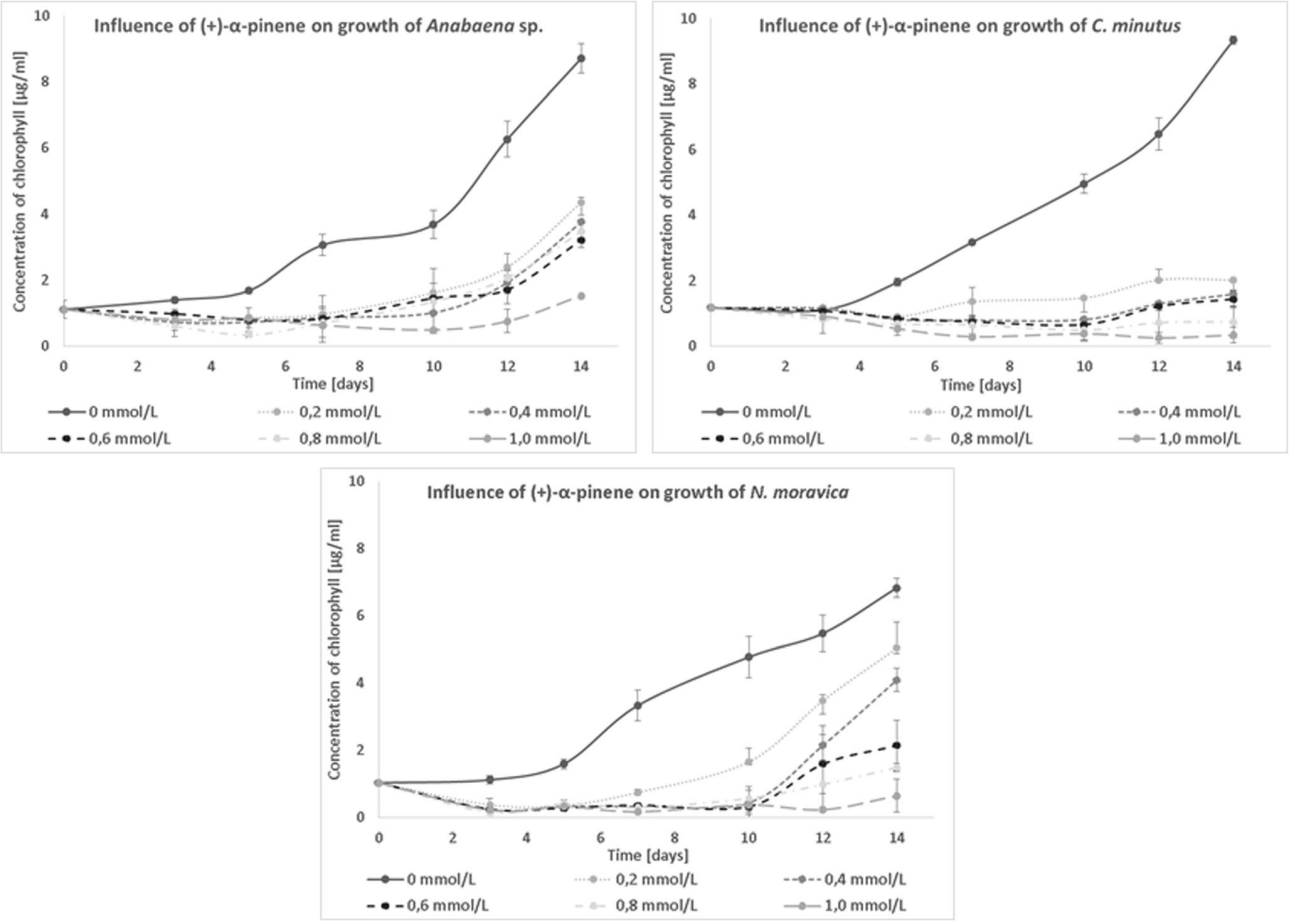
Influence of (+)-α-pinene on the growth of freshwater cyanobacteria

The influence of (+)-carvone was also investigated. This compound strongly inhibited the growth of all examined cyanobacteria. *Anabaena* sp. and *N. moravica* showed limited growth with all tested concentrations of this added compound, and their development was further reduced when the concentrations increased. The growth of *C. minutus* was inhibited so strongly that even with the lower concentration of (+)-carvone tested, after 3 days of incubation, no development was observed, and after 14 days of cultivation, the culture was dead (Fig. 5). (+)-Carvone was transformed to (+)-dihydrocarvone by all tested blue-green algae and to dihydrocarveol by *Anabaena* sp. (+)-Dihydrocarvone was a detoxification product. To study the influence of (+)-dihydrocarvone on cyanobacterial growth, an isomeric mixture of ~ 77% *n*-(+)-dihydrocarvone and ~ 20% iso-(+)-dihydrocarvone was used. This mixture had a smaller influence on the growth of *Anabaena* sp. and *C. minutus*. *N. moravica* developed similarly to how it did in the presence of (+)-carvone. These results showed that blue-green algae activated cell defense functions in an effort to transform more-toxic compounds into less harmful forms (Fig. 6).

(+)-α-Pinene reduced the development of *C. minutus*, *Anabaena* sp., and *N. moravica* at all tested concentrations. The most sensitive species was *C. moravica*—even the lower concentrations of (+)-α-pinene inhibited its development (Fig. 7).

**Fig. 8:**
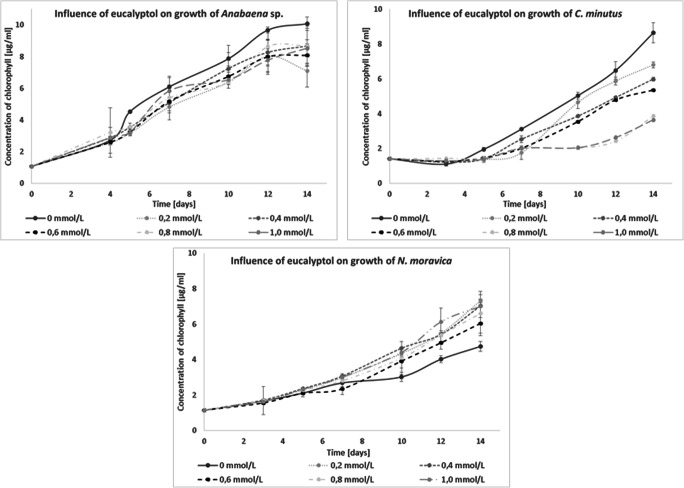
Influence of eucalyptol on the growth of freshwater cyanobacteria

Another tested compound was eucalyptol, which inhibited the growth of *C. minutus* and *Anabaena* sp. On the other hand, *N. moravica* was the only species whose growth increased with all tested concentrations of eucalyptol (Fig. 8).

**Fig. 9:**
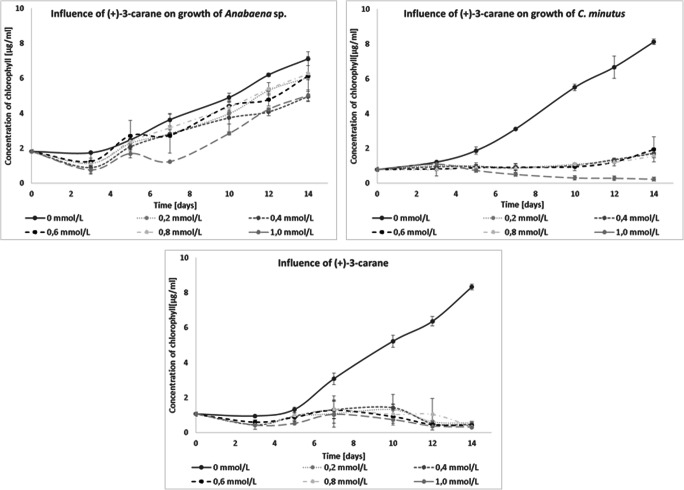
Influence of (+)-3-carane on the growth of freshwater cyanobacteria

In these tests, the best inhibitor of blue-green algae development was (+)-3-carene. It decreased the growth of *C. minutus* and *N. moravica* so strongly that very low chlorophyll contents were measured with all tested concentrations. *N. moravica* died after 14 days of culture. Only the development of *Anabaena* sp. was reduced by all tested concentrations of (+)-3-carene (Fig. 9).

### The influence of monoterpenoids on the growth of a cyanobacterial consortium

In this work, the term “cyanobacterial consortium” refers to a mixed culture of freshwater cyanobacteria, *Anabaena* sp., *C. minutus*, and *N. moravica*, where the total chlorophyll concentration on day 0 was 1 μg/mL, the result of combining of all three species such that each strain contributed 1/3 of the initial concentration of chlorophyll.

**Fig. 10:**
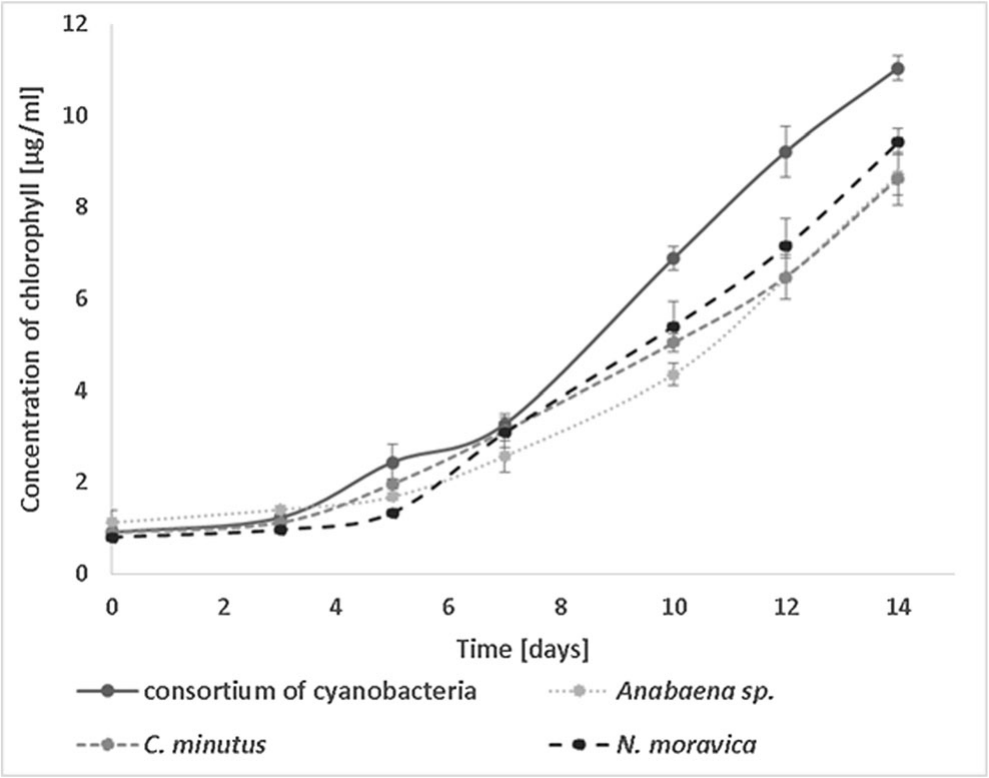
Growth of single freshwater blue-green algae and cyanobacterial consortium

The development of individual species of cyanobacteria and the consortium composed of these phototrophs is presented in Fig. 10. All three tested cyanobacterial species grew much better in their mutual presence—in the form of a consortium—than they did when cultured separately. This synergistic development enabled us to investigate the influence of selected monoterpenoids on the growth of the consortium comprising *Anabaena* sp., *C. minutus*, and *N. moravica*.

The development of a mixed culture of cyanobacteria in the presence of (+)-α-pinene was significantly reduced even at the lowest applied monoterpenoid concentration. Levels of (+)-α-pinene of 0.6, 0.8, and 1.0 mmol/L best restricted the growth of the cyanobacterial consortium. A comparison of data on the growth of individual species and the consortium with added (+)-α-pinene showed limited development of the mixture with increased monoterpenoid concentrations. An interesting result was obtained with *Anabaena* sp., as the species was less sensitive than were other cyanobacteria. Although microscopic observations showed the presence of each tested species, both in the control and with all concentrations of the studied monoterpenoid, the percentage change in the individual species in the consortium reflects their specific sensitivity to the tested compound. Notably, (+)-α-pinene more effectively inhibited the development of cyanobacteria, which, as in natural conditions, grow in multispecies consortia instead of individual isolates of the studied species (Fig. S2).

A citral concentration of 1.0 mmol/L in the cyanobacterial consortium culture led to the virtual death of the culture after 5 days. The data analysis presented in Figure S3 suggests that the higher the concentration of added monoterpenoid, the better the reduction. The cyanobacterial consortium was much more sensitive to citral than were the cultures of individual cyanobacteria. Only *N. moravica* exhibited similar properties in its growth individually and in the mixture. A very interesting result was obtained at a concentration 1.0 mmol/L citral. The consortium was virtually dead in this condition, but single cultures of *Anabaena* sp. and *C. minutus* were still normal. These results suggest antagonism in the mixture of cyanobacteria in the presence of citral. The microscopic observations conducted in this case also confirmed the presence of each species tested, both in the control and at all concentrations of the monoterpenoid. However, the consortium was more sensitive to citral than were the individual species that formed the culture.

Another tested compound was (±)-citronellal, which reduced the growth of the cyanobacterial consortium as the concentrations of this monoterpenoid increased. A (±)-citronellal concentration of 0.4 mmol/L resulted in significantly less growth than a concentration of 0.2 mmol/L. At this concentration, the development of the cyanobacterial mixture was practically identical to that of the control. The growth of freshwater cyanobacteria and their consortium with respect to the control with (±)-citronellal additive suggests that the development of a single species of *Anabaena* sp. is better than that of the cyanobacterial consortium with each of the tested concentrations. The growth of individual *N. moravica* and *C. minutus* cultures was considerably reduced up to approximately 80–90% over the control with (±)-citronellal. The results at the 1 mmol/L concentration showed a decrease in the development of the cyanobacterial consortium to approximately 10%, and this value was practically the same for *N. moravica* and *C. minutus* growth but differed significantly from that of *Anabaena* sp. The microscopic observations of the consortium showed the presence of each of the species tested, both in the control and with all concentrations of the monoterpenoid studied; the percentage change in the individual species in the consortium reflected their specific sensitivity to the test compound (Fig. S4).

(±)-Citronellol, similar to previously reported results, limited the development of the cyanobacterial consortium. Concentrations of 0.2, 0.4, and 0.6 mmol/L of this compound only slightly decreased cyanobacterial growth. The highest introduced concentration of (±)-citronellol essentially resulted in the inhibition of consortium growth by the 5th day of culture (Fig. S5). Cyanobacterial consortium development increased relative to that of the individual cyanobacteria in the presence of 0.2 and 0.6 mmol/L (±)-citronellol. The situation changed at a concentration of 1.0 mmol/L, where there was much better development of *Anabaena* sp. individually than in the consortium. At this concentration, the consortium was significantly inhibited. In addition, microscopic observations showed the presence of each of the examined species, both in the control and with all concentrations of the tested monoterpenoid.

The cyanobacterial consortium culture containing eugenol showed only slightly reduced development (Fig. S6). With low concentrations of eugenol, cyanobacterial development was similar to that under control cultures, whereas individual *Anabaena* sp. and *N. moravica* cultures developed more strongly than the controls. On the other hand, only the growth of *Anabaena* sp. was intensified. Interestingly, eugenol added even at the highest tested concentration did not limit the development of the cyanobacterial consortium as much as the other compounds did. The most sensitive species was *C. minutus*. The smaller dynamics of change in the consortium indicates the stabilizing role of interactions within the multispecies population in the presence of these external chemical inputs.

Similarly to the aforementioned eugenol, (+)-3-carene showed no ability to limit the development of the cyanobacterial consortium. Concentrations from 0.2 to 0.6 mmol/L mildly enhanced the development of the culture (Fig. S7). In comparing the consortium and individual cyanobacterial cultures, the consortium exhibited increased (+)-3-carene resistance, as was the case with eugenol. The simultaneous presence of all three tested species likely results in undefined synergy in response to the presence of this mono-oxidant. Microscopic images showed the presence of all cyanobacteria, both in the control and in test cultures with added (+)-3-carene.

Adding progressively more (+)-carvone to cultures of the cyanobacterial consortium progressively reduced growth up to approximately 60%. At the lowest of the tested concentrations, monoclonal cultures of *Anabaena* sp. and *N. moravica* showed increased development relative to the control, but as the (+)-carvone concentration increased, the cyanobacteria decreased in the consortium and in individual cultures. Notably, even then, *Anabaena* sp. and *N. moravica* grown separately developed better than they did in the consortium. These results suggest that this compound is a growth inhibitor in all types of tested cyanobacteria. Although microscopic observations showed the presence of each species tested, both in the control and at all concentrations of the monoterpenoid studied, the percentage changes in the individual species in the consortium reflected their specific susceptibility to (+)-carvone (Fig. S8).

### Biotransformation

None of the tested cyanobacteria measurably biotransformed citral, (±)-citronellal, (±)-citronellol, eugenol, (+)-α-pinene, eucalyptol, and of (+)-3-carene. Only (+)-carvone was transformed by all tested blue-green algae. The main product of this process was (+)-dihydrocarvone; only *Anabaena* sp. also produced (+)-dihydrocarveol. this is a surprising result of the lack of greater biotransformation capacity of monoterpene compounds, as literature data indicate such capacity (Balcerzak et al. 2014).

## Discussion

In this paper, we examined the influence of monoterpenoids, including citral, (±)-citronellal, (±)-citronellol, eugenol, (+)-carvone, (+)-dihydrocarvone, (+)-α-pinene, eucalyptol, and (+)-3-carene, on the growth of the freshwater cyanobacteria *Anabaena* sp*.*, *Chroococcus minutus*, and *Nodularia moravica.* We demonstrated that some monoterpenoids, such as α-pinene and (+)-carvone, can inhibit the growth of *Anabaena* sp*.*, *Chroococcus minutus*, and *Nodularia moravica.* The extent of the inhibitory effect depended on the concentration of the added compound and the type of species. *Nodularia moravica* was inhibited the best by (+)-3-carene, and eucalyptol increased the growth of this cyanobacterium.

*Chroococcus minutus* proved to be the most susceptible to the influence of the examined monoterpenes. It was strongly inhibited by (+)-carvone, (±)-citronellal, eugenol, (+)-α-pinene, and (+)-3-carene. However, citral, (−)-fenchone, and eucalyptol only slightly inhibited the growth of *Chroococcus minutus* when the concentration of the added monoterpenes increased.

*Anabaena* sp. displayed less growth when the concentration of added (+)-carvone, α-pinene, or (+)-3-carene increased. On the other hand, eugenol caused the accelerated growth of *Anabaena* sp. This species was the most resistant to the presence of the tested monoterpenoids.

Studies conducted on freshwater cyanobacterial consortia have demonstrated the synergism of the development of the mixture of *Anabaena* sp., *Chroococcus minutus*, and *Nodularia moravica*. In the case of this consortium, adding monoterpenoids limited development. Interesting results were obtained with (+)-3-carene, which, contrary to earlier results, led to the development of the cyanobacterial consortium. Most of the tested compounds inhibit the development of cyanobacteria, even when microorganisms develop in multispecies consortia, as in natural conditions, rather than in the individual species studied. Citral and (+)-α-pinene much more effectively inhibit the growth of cyanobacteria growing in the consortium than they do the growth of cultures with particular species.

To date, only the influence of essential oils and one monoterpen on the growth of cyanobacteria has been examined. Zhao et al. (2020) presented the physiological and molecular inhibition effect of eugenol on *Microcystis aeruginosa.* Eugenol had great potential for inhibition of the cell density of this cyanobacterium with increasing concentration (Zhao et al. 2020). There were obtained similar results for *Chroococcus minutus*. While the development of tested *Anabaena* sp. was not enhance in the presence of eugenol. This confirm our observation that the inhibitory activity of this compound largery depends on the type of examined cyanobacteria. Thus, Najem et al. (2016) reviewed the allelopathic activity of essential oils obtained from *Rosmarinus officinalis* L. on *Microcystis aeruginosa* and *Chroococcus minor* (Najem et al. 2016). In 2015, Wang et al. investigated the inhibitory effect of essential oils from six submerged macrophytes (*Potamogeton cristatus*, *Potamogeton maackianus*, *Potamogeton lucens*, *Vallisneria spinulosa*, *Ceratophyllum demersum*, and *Hydrilla verticillate*) on the growth of *Microcystis aeruginosa*, and the inhibition ratio was 30–40% (Wang et al. 2015); essential oils from *Arundo donax* and *Typha latifolia* were also investigated as growth inhibitors of these species of cyanobacteria (Wang et al. 2014). Tellez et al. (2000) reported that essential oils from *Callicarpa americana* are good inhibitors of the growth of *Oscillatoria perornata* (Tellez et al. 2000). Moreover, Zerrifi et al. (2020a, 2020b) investigated essential oils from seaweed (*Cystoseira tamariscifolia*, *Sargassum muticum*, and *Ulva lactuca*) against *Microcystis aeruginosa*, and only one of them, *Cystoseira tamariscifolia*, inhibited the growth of this cyanobacteria (Zerrifi et al. 2020a, 2020b). In another publication in 2020, Zerrifi et al. used essential oils from Moroccan plants (*Chenoporium ambrosioides*, *Thymus broussonetii*, *Thymus maroccanus*, *Thymus pallidus*, *Thymus satureioides*, *Satureja calamintha* L., and *Mentha suaveolens* L.) to investigate anticyanobacterial activity, and all of the studied EOs presented interesting activities against *Microcystis aeruginosa* (Zerrifi et al. 2020a, 2020b). Essential oils (EOs) are volatile, natural, complex compounds characterized by a strong odor and are synthesized by aromatic plants as secondary metabolites. Plants produce them to protect themselves against bacteria, viruses, fungi, insects, and herbivores by reducing their appetite for such plants. Essential oils are very complex and can contain as many as 60 components in different concentrations, but they are mainly characterized by two or three major components whose concentrations can account for as much as 70% of the EO. Terpenes and terpenoids are the main groups composing essential oils; for example, carvone makes up 58% and limonene makes up 37% the essential oils of the seed *Anethum graveolens* (Bakkali et al. 2008). Thus, in this article, we presented the interaction of individual terpenoids, and it can be concluded that individual components of essential oils are responsible for the inhibition or increased development of cyanobacteria.

In summary, the inhibitory effect depends on the types of added monoterpenoids and cyanobacteria. The best inhibitor and the most vulnerable cyanobacteria cannot be individually selected. Moreover, the present results can contribute to creating natural-origin products that will clear backyard ponds of bloom formations or call the attention of the cosmetic industry to use flavorings more carefully because they can pollute the water and enhance the growth of cyanobacteria.

## Supplementary Information

ESM 1 (PDF 577 kb)

## Data Availability

All data generated or analysed during this study are included in this published article.
