# Supplementary material for: Influence of monoterpenoids on the growth of freshwater cyanobacteria
Source: Appl Microbiol Biotechnol. 2021 Jun 23;105(13):5675–87. doi: 10.1007/s00253-021-11260-8 (PMC8285344; doi:10.1007/s00253-021-11260-8)
Supplement: Supplementary file 1 — (PDF 577 kb) [file 253_2021_11260_MOESM1_ESM.pdf]

Applied Microbiology and Biotechnology

Influence of monoterpenoids on the growth of freshwater cyanobacteria

Lucyna Balcerzak[1], Stanisław Lochyński[1, 2], Jacek Lipok[3]

[1] *Department of Chemical Biology and Bioimaging, Faculty of Chemistry, Wroclaw University of Science and Technology, Poland*
[2] *Institute of Cosmetology, Wrocław College of Physiotherapy, Wrocław, Poland*
[3] *Department of Pharmacy and Ecological Chemistry, Faculty of Chemistry, Opole University, Poland*

Correspondence :
Lucyna Balcerzak
e-mail: lucyna.balcerzak@pwr.edu.pl
Phone: (+48) (71) 320-20-10
Fax: (+48) (71) 320-24-27

Supplementary material

Content

Fig S1. Structures of monoterpenoids. 3
Fig S2. Influence on the growth of (+)-3-carane on single blue-green algae and their consortium.........4
Fig S3. Influence on the growth of (+)-α-pinene on single blue-green algae and their consortium. 4
Fig S4. Influence on the growth of citral on single blue-green algae and their consortium. 4
Fig S5. Influence on the growth of (±)-citronellal on single blue-green algae and their consortium. 4
Fig S6. Influence on the growth of (±)-citronellol on single blue-green algae and their consortium. 4
Fig S7. Influence on the growth of eugenol on single blue-green algae and their consortium. 4
Fig S8. Influence on the growth of (+)-carvone on single blue-green algae and their consortium..........4

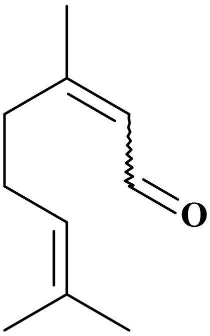

Citral

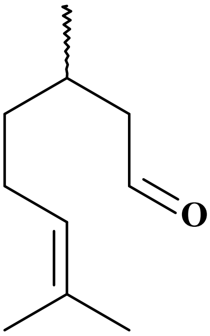

(±)-Citronellal

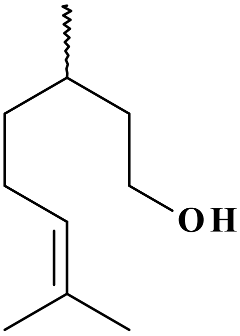

(±)-Citronellol

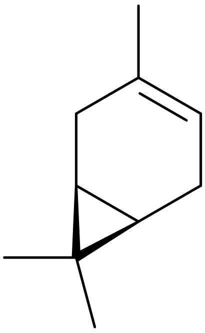

(+)-3-Carene

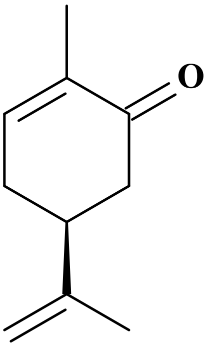

(+)-Carvone

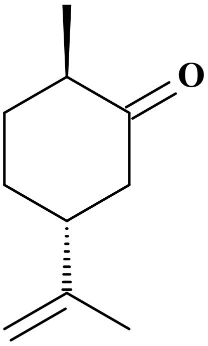

(+)-Dihydrocarvone

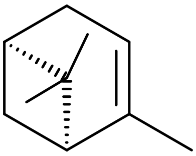

(+)-α-Pinene

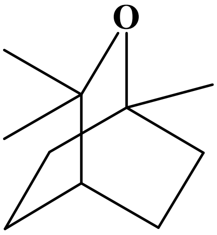

Eucalyptol

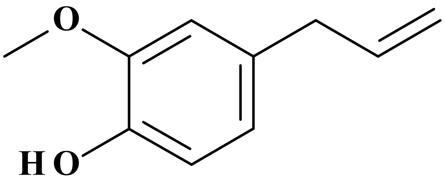

Eugenol

Fig S1. Structures of monoterpenoids.

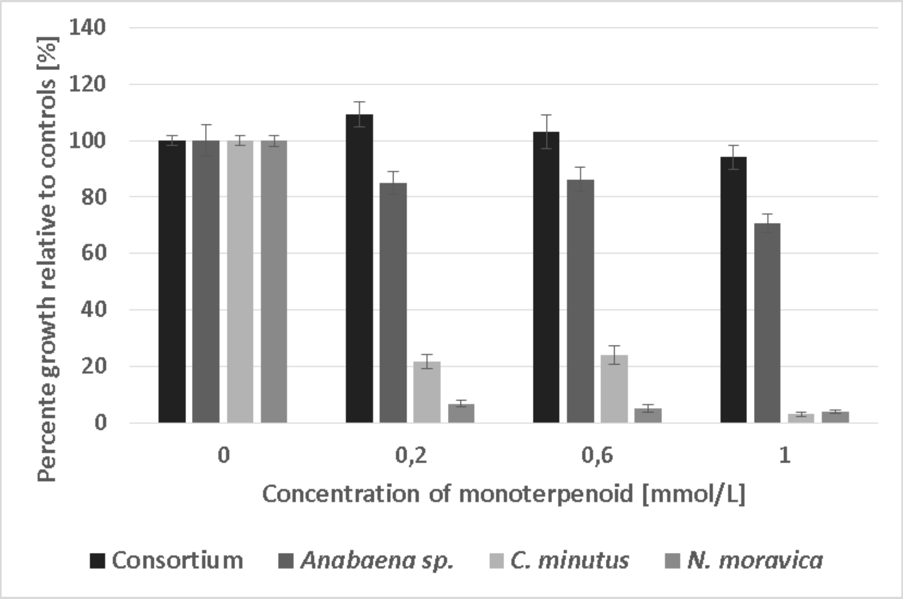

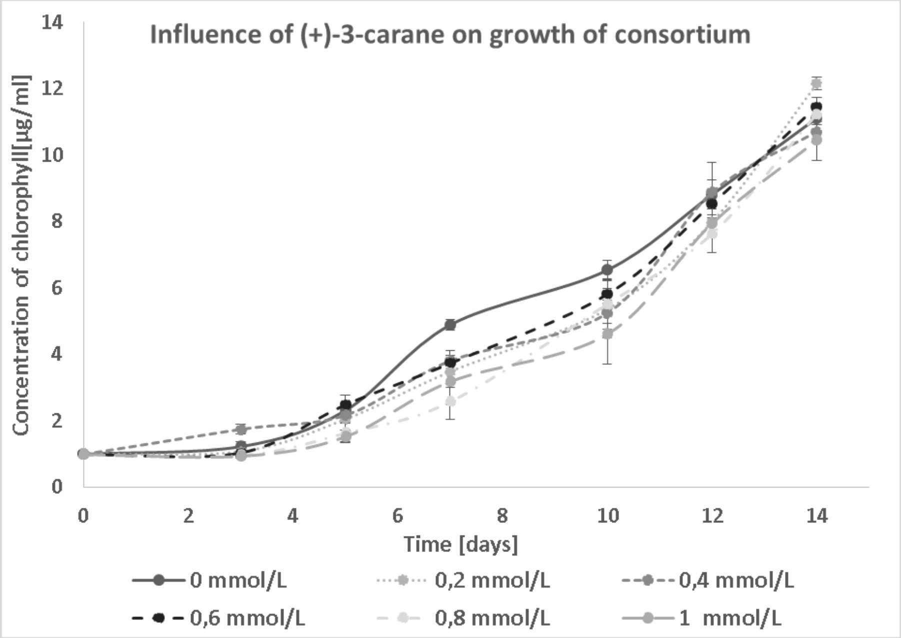

Fig S2. Influence on the growth of (+)-3-carane on single blue-green algae and their consortium.

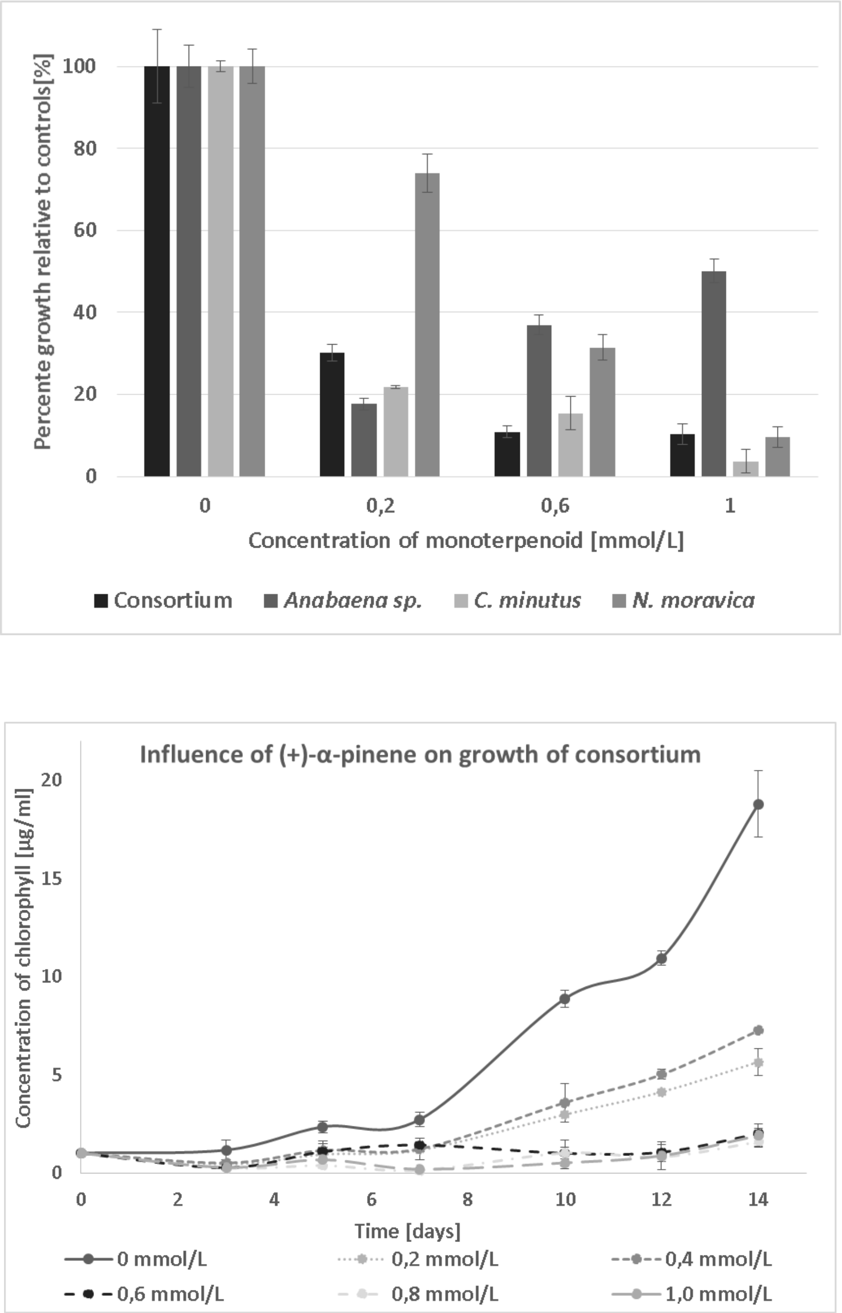

Fig S3. Influence on the growth of (+)-α-pinene on single blue-green algae and their consortium.

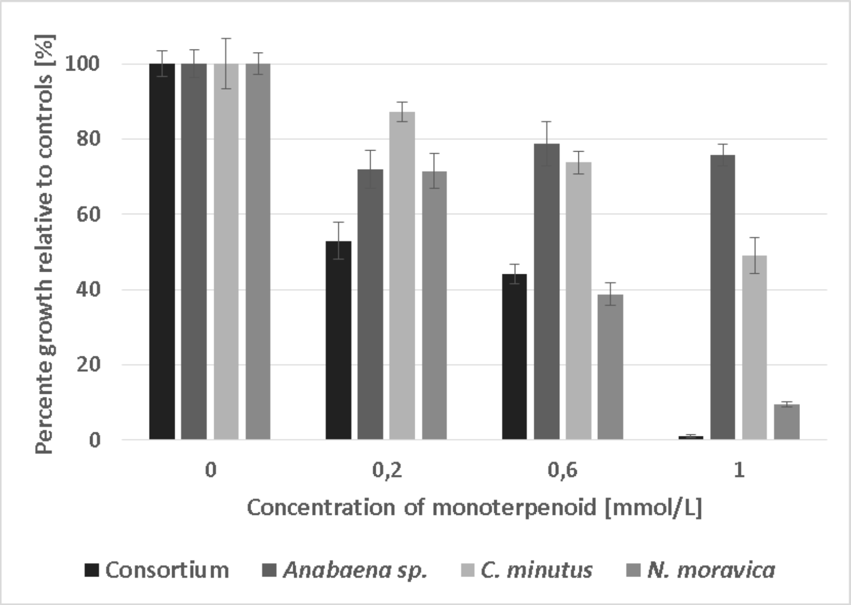

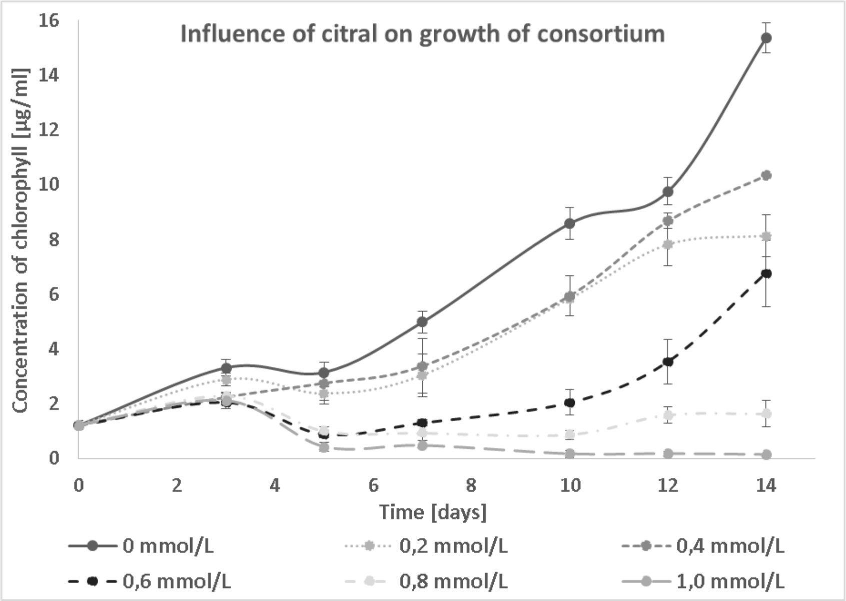

Fig S4. Influence on the growth of citral on single blue-green algae and their consortium.

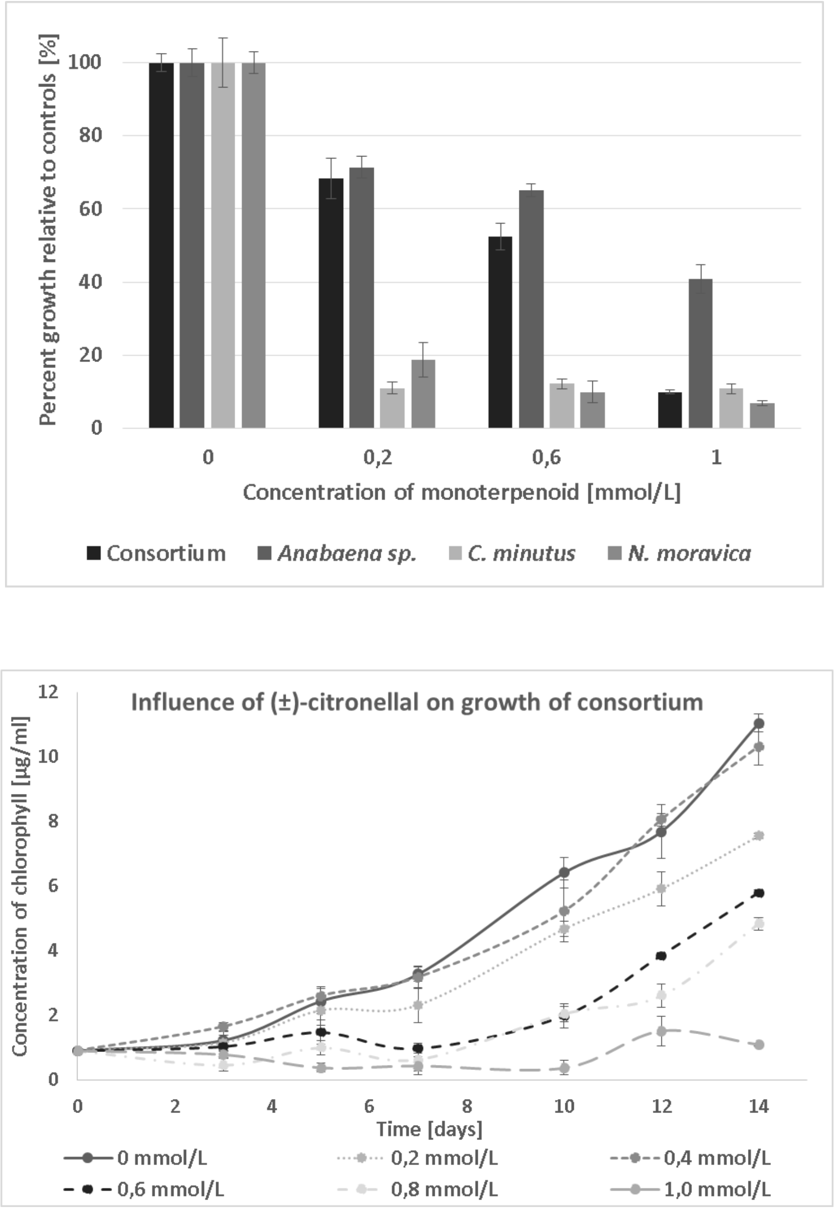

Fig S5. Influence on the growth of (±)-citronellal on single blue-green algae and their consortium.

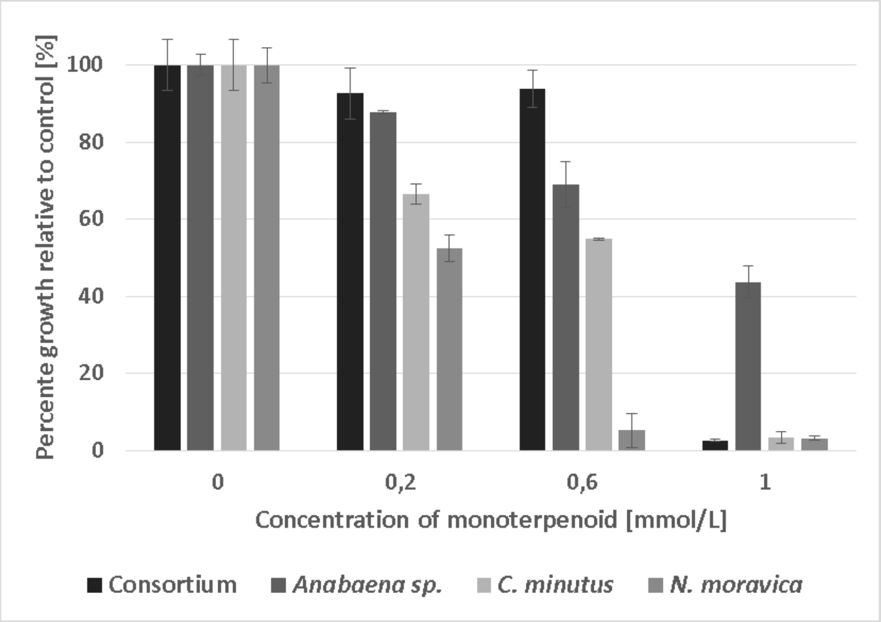

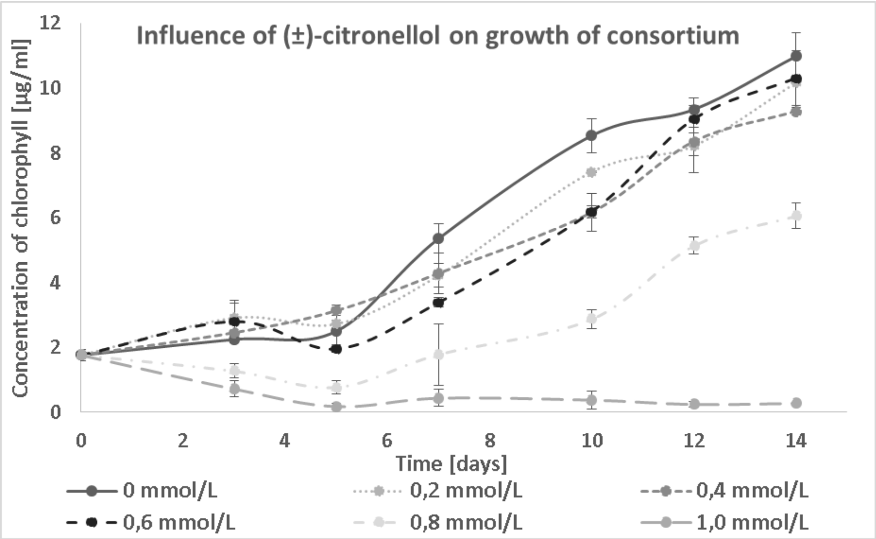

Fig S6. Influence on the growth of (±)-citronellol on single blue-green algae and their consortium.

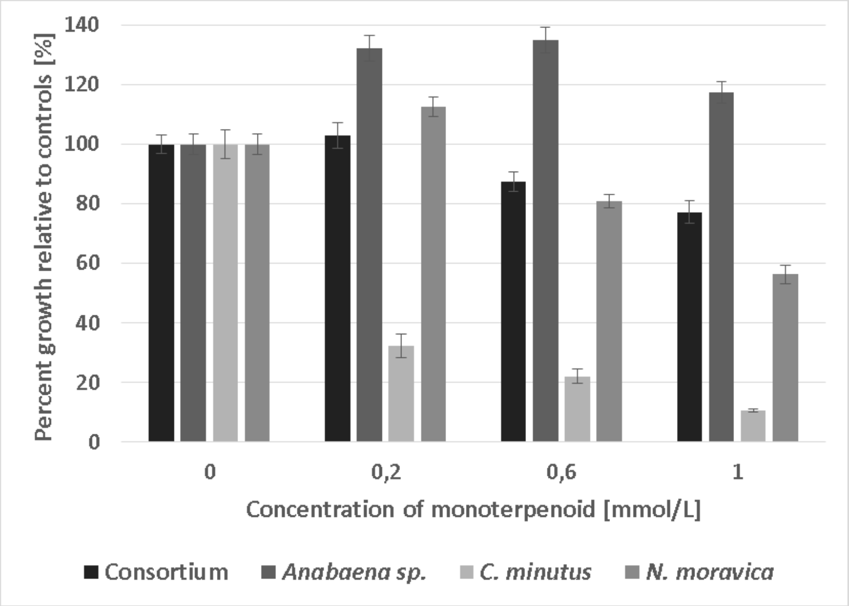

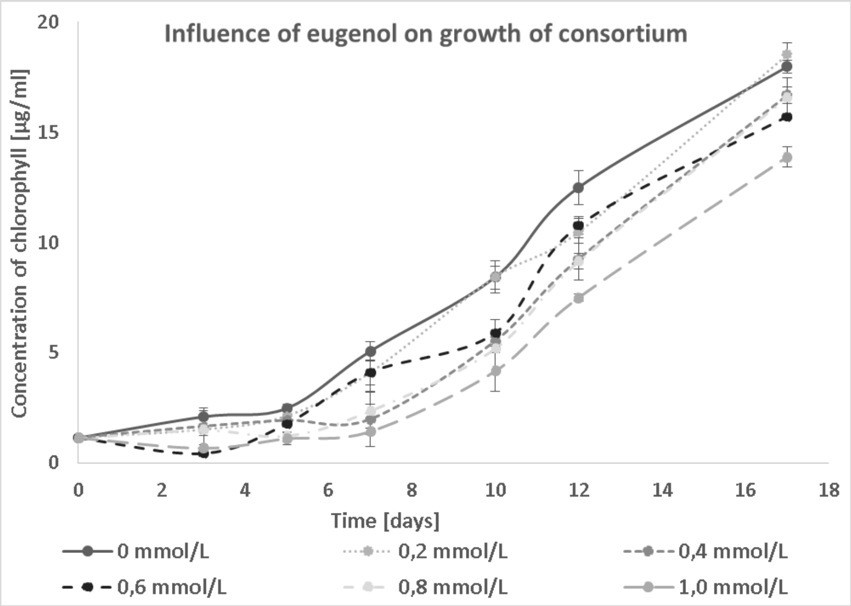

Fig S7. Influence on the growth of eugenol on single blue-green algae and their consortium.

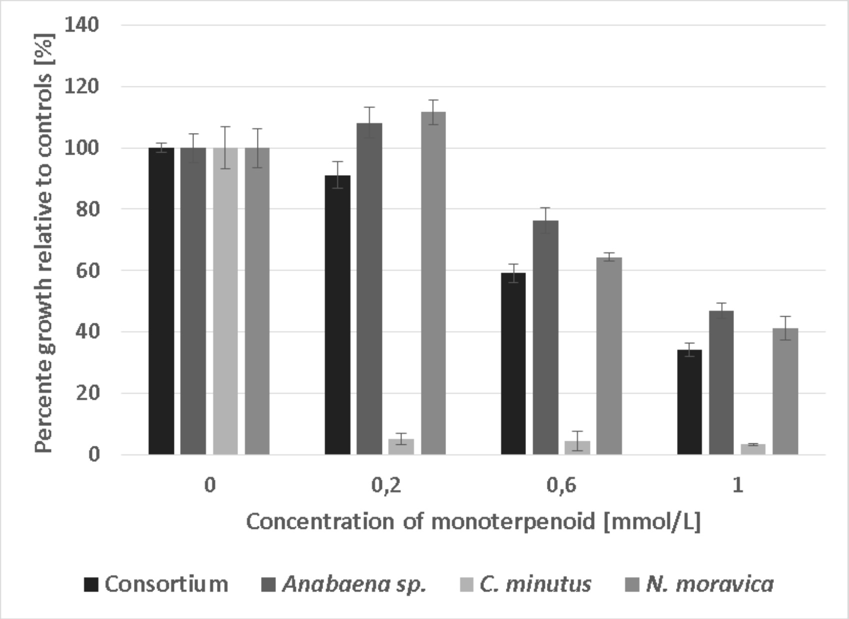

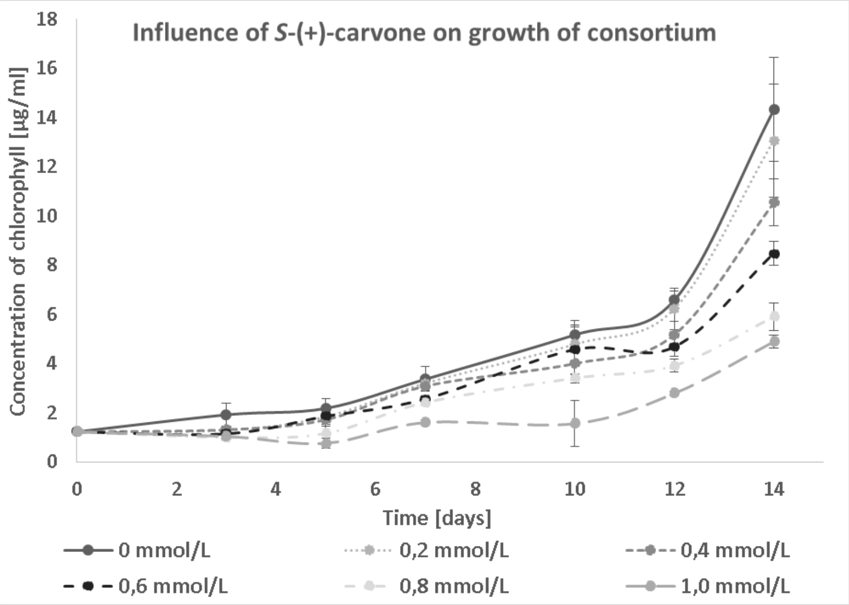

Fig S8. Influence on the growth of (+)-carvone on single blue-green algae and their consortium.
